# Primary effusion lymphoma occurring in the setting of transplanted patients: a systematic review of a rare, life-threatening post-transplantation occurrence

**DOI:** 10.1186/s12885-021-08215-7

**Published:** 2021-04-27

**Authors:** Magda Zanelli, Francesca Sanguedolce, Maurizio Zizzo, Andrea Palicelli, Maria Chiara Bassi, Giacomo Santandrea, Giovanni Martino, Alessandra Soriano, Cecilia Caprera, Matteo Corsi, Stefano Ricci, Linda Ricci, Stefano Ascani

**Affiliations:** 1 Pathology Unit, Azienda USL-IRCCS di Reggio Emilia, 42123 Reggio Emilia, Italy; 2 Pathology Unit, Azienda Ospedaliera Universitaria “Ospedali Riuniti” di Foggia, 71122 Foggia, Italy; 3 Surgical Oncology Unit, Azienda USL-IRCCS di Reggio Emilia, 42123 Reggio Emilia, Italy; 4 Clinical and Experimental Medicine PhD Program, University of Modena and Reggio Emilia, 41121 Modena, Italy; 5 Medical Library, Azienda USL-IRCCS di Reggio Emilia, 42123 Reggio Emilia, Italy; 6 Hematology Unit, CREO, Azienda Ospedaliera di Perugia, University of Perugia, 06121 Perugia, Italy; 7 Gastroenterology Unit, Azienda USL-IRCCS di Reggio Emilia, 42123 Reggio Emilia, Italy; 8 Pathology Unit, Azienda Ospedaliera Santa Maria di Terni, University of Perugia, 05100 Terni, Italy

**Keywords:** Lymphoma, Effusion, Epstein-Barr virus, Human Herpesvirus 8, Transplantation

## Abstract

**Background:**

Primary effusion lymphoma is a rare, aggressive large B-cell lymphoma strictly linked to infection by Human Herpes virus 8/Kaposi sarcoma-associated herpes virus. In its classic form, it is characterized by body cavities neoplastic effusions without detectable tumor masses. It often occurs in immunocompromised patients, such as HIV-positive individuals. Primary effusion lymphoma may affect HIV-negative elderly patients from Human Herpes virus 8 endemic regions. So far, rare cases have been reported in transplanted patients. The purpose of our systematic review is to improve our understanding of this type of aggressive lymphoma in the setting of transplantation, focusing on epidemiology, clinical presentation, pathological features, differential diagnosis, treatment and outcome. The role of assessing the viral serological status in donors and recipients is also discussed.

**Methods:**

We performed a systematic review adhering to the PRISMA guidelines. The literature search was conducted on PubMed/MEDLINE, Web of Science, Scopus, EMBASE and Cochrane Library, using the search terms “primary effusion lymphoma” and “post-transplant”.

**Results:**

Our search identified 13 cases of post-transplant primary effusion lymphoma, predominantly in solid organ transplant recipients (6 kidney, 3 heart, 2 liver and 1 intestine), with only one case after allogenic bone marrow transplantation. Long-term immunosuppression is important in post-transplant primary effusion lymphoma commonly developing several years after transplantation. Kaposi Sarcoma occurred in association with lymphoma in 4 cases of solid organ recipients. The lymphoma showed the classical presentation with body cavity effusions in absence of tumor masses in 10 cases; 2 cases presented as solid masses, lacking effusions and one case as effusions associated with multiple organ involvement. Primary effusion lymphoma occurring in the setting of transplantation was more often Epstein Barr-virus negative. The prognosis was poor. In addition to chemotherapy, reduction of immunosuppressive treatment, was generally attempted.

**Conclusions:**

Primary effusion lymphoma is a rare, but often fatal post-transplant complication. Its rarity and the difficulty in achieving the diagnosis may lead to miss this complication. Clinicians should suspect primary effusion lymphoma in transplanted patients, presenting generally with unexplained body cavity effusions, although rare cases with solid masses are described.

## Background

Primary effusion lymphoma (PEL) is a large B-cell lymphoma presenting as serous effusions in body cavities, usually without identifiable tumor masses, although very rare cases, presenting as tumor masses, so-called extracavitary PEL have been reported [1]. Currently, the presence of Kaposi Sarcoma Herpes Virus/Human Herpes Virus 8 (KSHV/HHV8) represents a diagnostic criterion for PEL [1]. PEL most often occurs in the setting of immunodeficiency and it is described predominantly in patients with HIV infection [1]. The occurrence of PEL in HIV-negative patients is uncommon; immunosuppression in these cases may be either a consequence of drugs, for instance following transplantation, or aging [1]. PEL is reported in older people from Human Herpes Virus 8 (HHV8) endemic geographic areas. The epidemiology of PEL is very similar to Kaposi sarcoma (KS). PEL predominantly affects males and most often arises at younger age in HIV-infected individuals compared to HIV-negative patients (42 vs 73 years) [1]. PEL arising in HIV-positive individuals is usually coinfected by HHV8 and Epstein Barr virus (EBV), whereas in HIV-negative cases, PEL may lack EBV infection [1].

Very few cases of PEL have been reported in the setting mainly of solid organ transplant (SOT) recipients, with only one case after allogenic bone marrow transplantation [2–13].

Our purpose was to achieve a better understanding of post-transplant PEL (PT-PEL) through an extensive systematic literature review, focusing on knowledge gained in terms of epidemiology, clinical presentation, timing from transplant to PEL diagnosis, histopathological and molecular features, treatment and prognosis. The role of assessing HHV8 serological status in donors and recipients and the differential diagnosis with the other HHV8-related lymphoproliferative diseases is also discussed.

## Methods

Adhering to the Preferred Reported Items for Systematic Reviews and Meta-Analyses (PRISMA) guidelines, we performed a systematic literature review. The search was conducted using PubMed/MEDLINE, EMBASE, Scopus, Cochrane Library (Cochrane Database of Systematic Reviews, Cochrane Central Register of Controlled Trials-CENTRAL) and Web of Science (Science and Social Science Citation Index) databases, with the non-MeSH/MeSH terms “primary effusion lymphoma” AND “post-transplant” [Mesh]. The search was performed from the inception of the databases until November 30, 2020. The criteria for inclusion were as follows: 1) a diagnosis of PEL based on reliable morphological and immunophenotypical features according to current criteria of World Health Organization (WHO) Classification of Tumours of Haematopoietic and Lymphoid Tissues [1]; 2) PEL arising in the setting of transplanted patients; 3) retrospective, observational case-control studies, case reports and/or series, literature review. The exclusion criteria were as follows: 1) studies not published in English; 2) lack of adherence to the diagnostic criteria for PEL according to current WHO classification [1]. Three independent reviewers (ZaM, SF, ZiM) identified papers on the basis of title, abstract and key words; then they ascertained whether the selected papers met the inclusion criteria by reading the article full-texts. Reference lists from each retrieved article were further checked in order to find additional reports. From those selected papers, the following information was collected: author’s surname; year of publication; patient’s age and sex; ethnicity; serological status; type of transplant and timing from transplant to PEL diagnosis; type of immunosuppressive therapy at time of PEL diagnosis; presence of Kaposi sarcoma and/or other malignancies and timing from transplant; PEL location; morphological, immunohistochemical and molecular features of PEL; therapy; duration of follow-up and clinical outcome. A fourth independent reviewer (AS) revised all collected results and resolved eventual discrepancies. Finally, this search identified an overall number of 12 articles [2–13].

## Results

### Epidemiology

To date, 12 studies have analyzed PT-PEL, with a total of 13 cases. Patient characteristics, in addition to morphological, immunohistochemical and molecular features and clinical data, including treatment and outcome, are summarized in Table 1.

**Table 1:** Clinicopathological features of primary effusion lymphoma in post-transplant patients

Ref/ age, sex, ethnicity/	Serology/ possible source of HHV8 infection	Transplanted organ/time from TP to PEL diagnosis	IS therapy/HAART therapy	KS or other malignancies/pre-TP or post-TP	Site of PEL/BM involvement	Histology	IIC/EBER	Molecular data	Therapy survival from PEL diagnosis
**Jones 1998** **59/M/Haitian**	HIV-; no drug abuse; no sex with men	Heart/ 94 mos	AZA + CYA+ prednisone	KS (5 mos after TP)	Bilateral pleural effusions/BM NA	Large plasmacytoid cells, moderate basophilic cytoplasm, prominent multiple nucleoli	CD30+ EMA+ CD38+ CD10- CD19- CD20- CD79α- CD3- CD5- CD45RO- CD11b- CD13- CD14- CD33- CD45- CK- EBVLMP1- EBER-	IGH+,HHV8 DNA + EBVDNA-	CYA reduction Cycloph+ VCR+ prednisone (1cy). Bleo. CHOP (1cy). Ifo + eto (2 cy). Death 6 mos later.
**Dotti 1999** **56/M/unknown**	HCV+ HIV -HBV-	Heart/42 mos	AZA+ CYA + steroid	No	Peritoneal effusion BM positive at molecular analysis	Medium, large-sized cells, abundant basophilic cytoplasm, convoluted nuclei, large and multiple nucleoli	CD45+ CD38+ CD138+ HLA-DR+ CD30 + CD34- CD13-CD33- CD3-CD2- CD5-CD7- CD10-CD20- CD19-CD56- κ- λ-	IGH+ HHV8 DNA integration EBV genome integration 11q23 deletion No BCL6 c-MYC ALL-1 rearrangements. No Bcl2/IGH translocation.	CYA reduction, AZA stopping. No CT (for poor performance status). Death 1 mo later
**Boulanger 2008** **57/M/African**	HIV- HHV8+ (before TP)	Kidney/44 mos	TC+ MMF+ prednisone. 5 mos after TP: KS progression despite MMF stopping, TC and prednisone reduction+CT (dauno+ docetaxel+ bleo) 24mos after KS onset: TC stopping+ RAPA with partial KS remission. At PEL diagnosis RAPA blood level 12 ng/ml	Disseminated KS (5 mos after TP)	Right pleural effusion BM NA	PEL morphology	CD138+ CD38+ HHV8+ EBER- CD3- CD20-	IGH+ TCR-HHV8 DNA+ (PCR) Oligoclonal HHV8 episomes+	CHOP + bleo (4 cys) Death 8 mos later
**Boulanger 2008** **63/M/African**	HIV- HHV8+ (before TP)	Kidney/54 mos	CYA+ MMF+ prednisone+ Anti CD25 moAb 3 mos after TP Meth (for rejection). 14 mos after TP CYA converted to RAPA (for renal graft impairment). At PEL diagnosis RAPA blood level 8.5 ng/ml	No	Cardiac tamponade BM NA	PEL morphology	CD30+ CD138+ HHV8+ EBER- CD20- CD3-	IGH- TCR-Oligoclonal HHV8 episomes+	Cidofir Death 1 mo later
**Melo 2008** **67/M/unknown**	NA	Kidney/132 mos	AZA+ CYA+ prednisone	No	Left pleural effusion BM NA	Large hyperbasophilic cells with multiple nucleoli	CD45+ CD38+ CD138+ Negativity for B and T cells markers	HHV8+ EBV+ (PCR)	Cycloph+VCR + prednisone CY and AZA stopped RAPA (blood level 7 ng/ml)
**Testa 2010** **55/M/unknown**	HIV- HBV- HCV- HAV-CMV-HSV2-EBVDNA-HBVDNA- HCVRNA-CMVDNA-HSVDNA-HHV8-	Liver/12 mos	TC + MMF	No	Massive neoplastic infiltration of lungs, pleura, heart, IC muscles, around large vessels, Gerota fascia, omentum. Sclerosing peritonitis of stomach, intestine, liver BM NA	Large cells, prominent nucleoli, abundant basophilic cytoplasm	CD138+ EMA+ CD43+ vimentin+ HHV8+ kappa/lambda polyclonal ki67 > 90% CK- CD20- CD3- CD45- EBVLMP1- CD56- CD57- MPO- CD34- Melan-A- CD99- Desmin- CD68-TdT- CD30- calretinin-	HHV8DNA + EBVDNA- HSVDNA- CMVDNA- HHV6-	Diuretics Death 4 mos later Autopsy performed
**Rose 2012** **42/M/Italian**	HIV- HBV- HCV-	ASCT/276 mos	Myeloablative conditioning (Cicloph+TBI); chronic GVHD treated with CYA prednisolone, PUVA, THA. 24 mos after TP: pericarditis; THA stopped	No	Marked peritoneal effusion, small pleural effusion. Constrictive pericarditis BM NA	Atypical lymphoid cells	CD45+ CD138+ TdT- CD34- MPO- CD3- CD5- CD20- CD79α-CD30- ALK1-Ki67 100%	PCR: HHV8+	PericardiectomyDeath 2 weeks later
**Shi 2012** **60/M/Chinese**	Serology pre-transplant: NA Serology at PEL diagnosis: HIV- HHV8+	Kidney/120 mos	Long-term IS therapy (NS)	No	Bilateral pleural effusion, peritoneal effusion BM negative	Large cells with large nuclei, prominent nucleoli, scanty cytoplasm	HHV8+ CD30+ CD45+ vimentin+ EMA+ CD3- CD15- CD20- CD43- CD79α-CD45RO- CK-ALK1- EBVLMP1-	IGH+ IGL+	CT (endoxan, farmorubicin, oncovin, prezolon). Death 4 mos later
**Christenson 2015** **72/M/unknown**	HIV- EBV DNA- CMV-No drug abuse; no sex with men; no link with HHV8 endemic areas	Liver/120 mos	TC (119 mos) changed to RAPA 1 mo prior PEL (due to altered renal function)	SCC of head and neck (surgery, CT, RT)	Pleural effusion BM NA	Large plasmacytoid cells, large convoluted nuclei, prominent nucleoli	CD45+ CD30+ CD38+ MUM1+ HHV8+ EBER -	NA	Rapamycin maintained+pleural drainage+intrapleural cidofovir. Due to cidofovir intolerance, bortezomib+Doxo. Death 7 mos later
**Kalogeraki 2015** **49/M/unknown**	HIV-	Kidney/340 mos	Prolonged IS therapy	No	Peritoneal effusion BM NA	Large cells with high N/C ratio, pleomorphic nuclei, prominent nucleoli, amphophilic cytoplasm	CD3+ CD138+ PAX5+ CD30+ CD45+/−HHV8+ (in situ hybridization) EBER-CK- EMA- CD2- CD5- CD19- CD20- CD43- CD79α- ALK1- TIA1-EBVLMP1-	IGH+ TCR+	CHOP Alive at 10 mos
**Cain 2018** **29/M/unknown**	HIV + HBV- HCV – CMV-	Kidney/24 mos	TC + HAART	Nodal KS identified at autopsy	Pleural effusion lymph nodes spleen liver heart kidney lung BM NA	Intravascular large plasmacytoid and immunoblastic cells	CD45+ MUM1+ HHV8+ EBER+ CD20- CD79α- CD19 PAX5- CD2- CD3- CD5- CD7- CD4- CD8- ALK- MPO- CD138- kappa- lambda- IgM- IgD- CD34- CD117- CD30- EMA- CD10- Bcl6- TdT-	IGH and TCR NA for poor DNA quality	Death 2 weeks after admission Autopsy performed
**Kugasia 2018** **63/M/Haitian**	Remote history of polysubstance abuse Pre-transplant: HIV- EBV- HHV8- Post-transplant: HIV- EBV- HHV8+	Heart/5 mos	TC+ prednisone	Cutaneous KS (pre-TP) treated with RT. KS recurrence at PEL diagnosis	Left pleural effusion BM positive	Large atypical cells with high N/C ratio, prominent nucleoli	CD45+ CD30+ HHV8+ CD138+ MUM1+ CD20- PAX5- CD4- CD8- CD56- ALK1- EBVLMP1- EMA- BCL2- BCL6- cyclin D1- CD10- calretinin -WT1- Ber-EP4-	NA	Cycloph, VCR, prednisone, brentuximab TC changed to sirolimus. (HHV8 undetectable) Death 14 mos later for acute graft rejection
**Zanelli 2019** **42/M/Italian**	HIV- HHV8- EBV-	Small bowel/7 mos	TC+ Anti-CD52 moAb	No	Multiple gastric and duodenal polyps BM negative	Large cells with eccentric nuclei and prominent nucleoli	CD138+ CD38+ CD30+ HHV8+ EBER+ Ki67 70% EMA+ lambda+ CD20- CD79α- PAX5- CD3- CD5-	NA	Death 1 mo later

*Legends*: *ASCT* allogenic stem cell transplant, *Aza* azathioprine, *bleo* bleomycin, *BM* bone marrow, *CHOP* cyclophosphamide, doxorubicin, prednisone, vincristine, *Cy* cycle, *CYA* cyclosporine, *Cys* cycles, *Cycloph* cyclophosphamide, *CT* chemotherapy, *Dauno* daunorubicin, *Doxo* doxorubicin, *EBV* Epstein Barr virus, *Eto* etoposide, *GVHD* graft versus host disease, *Ifo* ifofosfamide, *IGH* immunoglobulin heavy chain, *IGL* immunoglobulin light chain, *IS* immunosuppressive, *KS* Kaposi sarcoma, *Meth* methilprednisolone, *MMF* mycophenolate mofetil, *MoAb* monoclonal antibody, *Mo* month, *Mos* months, *NA* not available, *NP* not performed, *NS* not specified, *PEL* primary effusion lymphoma, *Rapa* rapamycin, *RT* radiotherapy, *TBI* total body irradiation, *TC* tacrolimus, *THA* thalidomide, *TP* transplant, *VCR* vincristine

Patients with PT-PEL were aged between 29 and 72 years, with an average age of 54.9 years. Patients in their fourth, fifth and sixth decade of life were the most commonly affected. All PT-PEL occurred in males. In 7/13 cases ethnicity was reported (2 Haitian; 2 African; 2 Italian; 1 Chinese). In most cases (12/13) PEL developed after solid organ transplant (SOT) and only in one patient the disease presented after allogenic stem cell transplantation (ASCT) [7]. In 6/12 post-SOT cases, PEL occurred after kidney transplant [4, 5, 8, 10, 11]; in 3/12 cases, after cardiac transplant [2, 3, 12]; in 2/12, after liver transplant [6, 9]; in one case, after small bowel transplant [13]. The time from transplant to PEL diagnosis was between 5 to 340 months, with a median of 97.6 months.

### Serological status and possible sources of HHV8 infection

Patients were HIV-negative in the majority of cases with known serological status (11/12), only one PT-PEL occurred in an HIV-positive patient [11]. HHV8 serology was reported in 6/13 cases. Two/6 patients, both from endemic regions, were pre-transplant HHV8-seropositive [4]. Two/6 patients (one of unknown ethnicity and the other from an HHV8 endemic area) resulted HHV8-negative at pre-transplant serological screening [6, 13]. In one patient from a non-endemic area, pre-transplant HHV8 serological status was not available and the patient resulted HHV8- seropositive at PEL diagnosis [8]. One patient, from an endemic area, was pre-transplant HHV8-negative and resulted HHV8-positive after transplant [12]. In the last patient, a remote history of polysubstance abuse was reported.

### Immunosuppressive treatment following transplantation

At time of PEL diagnosis, all patients were receiving prolonged immunosuppressive treatment following transplantation. Azathioprine, cyclosporine and steroid in combination were used in 3/11 cases with known immunosuppressive treatment [2, 3, 5]. Tacrolimus was administered in 6/11 cases as follows: tacrolimus alone for 119 months after liver transplant and changed to rapamycin 1 month prior to PEL diagnosis (1/6) [9]; tacrolimus in combination with mycophenolate mofetil (1/6) [6], prednisone (1/6) [12], anti-CD52 antibody (1/6) [13], Highly Active Anti-Retroviral Therapy (HAART) (1/6) [11] or mycophenolate mofetil plus prednisone (1/6) [4]. In the last case, tacrolimus was changed to rapamycin for KS progression. In 1/11 cases cyclosporine was used associated with mycophenolate mofetil, prednisone and anti-CD25 antibody; then cyclosporine was converted to rapamycin for renal graft function impairment, 40 months before PEL diagnosis [4]. In the case occurring after ASCT, the patient had received myeloablative conditioning with cyclophosphamide and total body irradiation, followed by cyclosporine, steroid and thalidomide for graft versus host disease (GVHD) [7].

### PEL clinical presentation

PT-PEL presented as serous effusions in body cavities in 10/13 cases (pleural effusion: 5/10 [2, 4, 5, 9, 12]; peritoneal effusion: 2/10 [3, 10]; pleural and peritoneal effusions: 2/10 [7, 8]; pericardial effusion: 1/10 [4]). In 1/13 cases, PT-PEL presented as pleural effusions associated with multiple organ involvement [11]. Two/13 cases were considered to be the extracavitary variant of PEL, as the patients had solid masses without any involvement of body cavities [6, 13]; in one of these cases, the lymphoma involved lungs, pleura, heart, intercostal muscles, Gerota fascia and omentum [6]; in the other case, the disease presented as multiple gastric and duodenal polyps [13]. Bone marrow biopsy was positive in 2/4 cases evaluated [3, 12]; in one of these 2 cases bone marrow involvement was identified only at molecular level, being present a clonal neoplastic population [3].

### Malignancies other than PEL

In 4/13 cases, KS occurred [2, 4, 11, 12]. In 2/4 cases, KS preceded PEL, developing 5 months after transplant [2, 4] and in one of these, KS initially progressed despite chemotherapy and reduction of immunosuppressive treatment (mycophenolate mofetil stopping and tacrolimus plus prednisone reduction); then KS partial remission was obtained with rapamycin administration and tacrolimus stopping [4]. In 1/4 cases, KS was discovered at post-mortem examination [11], whereas in 1/4 cases, KS occurred before transplant and was successfully treated with radiotherapy, but it recurred at PEL diagnosis [12]. In the case reported by Christenson, the patient developed squamous cell carcinoma of head and neck and was treated with surgery plus chemotherapy and radiotherapy [9].

### Microscopic and immunohistochemical findings

PT-PEL was mostly diagnosed on the basis of cytological smears and cell block sections of effusion fluid. The cells were large, often pleomorphic, with prominent nucleoli and abundant basophilic cytoplasm occasionally showing vacuoles. The cells resembled immunoblasts, plasmablasts or anaplastic cells sometimes with Reed-Sternberg-like features. In the case by Cain et al [11], in which pleural effusions were associated with multi-organ involvement, histology revealed a peculiar intravascular pattern of growth. The lymphoma cells usually expressed CD45, markers of lymphocyte activation (CD30, CD38, EMA, HLA-DR) and markers of plasma cell differentiation (CD138, MUM1/IRF4). B-cell (CD19, CD20, CD79α) and T-cell (CD3, CD4, CD8) markers were usually absent. Aberrant CD3 positivity, in absence of other T-cell marker expression, was observed in the case by Kalogeraki et al [10]. The extracavitary-variant of PEL may be slightly different from classic PEL, being more often positive for some B-cell markers and negative for CD45/LCA. However, both cases of extracavitary PT-PEL were found to be negative for B-cell markers [6, 13]. PEL diagnosis requires the detection of HHV8 in the neoplastic cells. The standard assay to demonstrate viral infection is usually immunohistochemistry, detecting expression of HHV8-encoded latency-associated nuclear antigen 1 (LANA-1) protein. Epstein-Barr virus (EBV) infection was found by in situ hybridization for EBV-encoded small RNA (EBER) only in 2/7 cases evaluated [11, 13], one of which occurred in an HIV-positive individual [11]. Immunohistochemical assay of EBV latent membrane protein 1 (LMP1) was negative in 5/5 tested cases [2, 6, 8, 10, 12].

### Molecular data

Polymerase chain reaction (PCR) demonstrated the presence of HHV8 genome in 7/7 evaluated cases [2–7], two of which were tested by immunohistochemistry and found to be HHV8-LANA-1 positive [4]. EBV was identified by PCR in 2/4 cases, in which EBER was not tested [3, 5]. Clonal immunoglobulin heavy chain (IGH) gene rearrangement was identified in 5/7 cases evaluated [2–4, 8, 10] and in one of these, clonal immunoglobulin light chain (IGL) gene rearrangement was also identified [8]. In 1/7 cases, IGH gene rearrangement was negative [4] and in 1/7 cases, the result was not conclusive for poor DNA quality [11]. T-cell receptor (TCR) gene rearrangement was identified in 1/4 cases tested, the case by Kalogeraki et al [10] in which aberrant CD3 immunohistochemical expression was observed; TCR gene rearrangement was negative in 2/4 cases [4] and in 1/4, TCR result was not available for poor DNA quality [11]. Dotti et al identified the presence of 11q23 deletion, whereas BCL2/IGH translocation as well as BCL6, c-MYC and ALL-1 rearrangements were absent [3].

### Treatment and outcome

In 7/13 cases, different chemotherapeutic schemes were performed (1/7: CHOP (cyclophosphamide, doxorubicin, prednisone, vincristine) [10]; 1/7: CHOP plus bleomycin [4]; 1/7: CHOP plus bleomycin and ifofosfamide plus etoposide [2]; 1/7: cyclophosphamide plus vincristine plus prednisone [5]; 1/7: endoxan plus farmorubicin plus oncovin plus prezolon [8]; 1/7: intrapleural cidofovir, then, due to citofovir intolerance, bortezomib plus doxorubicin [9]; 1/7: cyclophosphamide plus vincristine plus brentuximab [12]. In 1/13 cases, only antiviral treatment with cidofovir was used [4]. Changes in the immunosuppressive treatment were performed at PEL diagnosis in 4/13 cases as follows: 1/4: tacrolimus was changed to sirolimus (rapamycin) resulting in undetectable HHV8 DNA blood level [12]; 1/4: cyclosporine reduction [2]; 1/4: cyclosporine reduction plus azathioprine stopping [3]; 1/4: cyclosporine and azathioprine stopping plus introduction of rapamycin [5]. In 1/13 cases, tacrolimus was changed to rapamycin due to altered renal function, 1 month prior PEL diagnosis and then rapamycin was continued [9]. Eleven/12 patients with available follow-up died from 2 weeks to 14 months from PEL diagnosis. In the case by Kugasia et al, death occurred 14 months after PEL diagnosis and it was unrelated to lymphoma [12]. Only 1/12 was alive at 10 months from PEL diagnosis, undergoing CHOP regimen [10].

### Differential diagnosis

The main differential diagnoses for PEL include a spectrum of HHV8-associated lymphoproliferative disorders such as HHV8-positive multicentric Castleman disease (MCD) [1], HHV8-positive diffuse large B-cell lymphoma, not otherwise specified (HHV8-positive DLBCL, NOS) [1] and germinotropic lymphoproliferative disorder (GLPD) [1, 14]. Diffuse large B-cell lymphoma associated with chronic inflammation (DLBCL-CI) and HHV8-negative effusion-based lymphoma (EBL) need also to be considered in the differential diagnosis. The main differential diagnoses are summarized in Table 2.

**Table 2:** Main differential diagnoses

	PEL	HHV8+ MCD	HHV8+ DLBCL	GLPD	DBLCL-CI	HHV8-negative EBL	PBL
**Immunodeficiency**	Usually present	Mostly present	Mostly present	Absent	Local immunodeficiency from longstanding chronic inflammation in a restricted space	Absent	Mostly present (HIV-related or due to age, transplant, autoimmune diseases or iatrogenic)
**HIV serology**	+ (− in elderly and EBV-negative cases)	+ (90% of cases)	+ (almost always)	- (rarely +)	–	–	+ (often)
**Age/Sex/Outcome**	Adults, mainly males (HIV-negative pts. are older) Unfavorable	Adults; HIV+ pts. mainly males Unfavorable	Adults Unfavorable	Adults Often favorable	Adults Unfavorable	Pts older than PEL pts. Unfavorable	Adults (commonly) Unfavorable
**Clinical presentation**	Effusion (classic PEL). Extra-nodal sites (often) and lymph nodes in extracavitary PEL.	Multiple lymphadenopathy, splenomegaly, KS, systemic symptoms	Systemic disease (nodal and extranodal sites, spleen, BM)	Lymphadenopathy (usually isolated)	Tumor mass involving body cavities or narrow spaces	Effusion (without detectable tumor masses as classic PEL)	Extranodal sites; rarely lymph nodes
**Association with MCD**	rare	+	frequent	–	–	–	–
**Histology**	PB/IB generally in fluids	PB/IB (single or in small aggregates mostly in mantle and interfollicular zones)	PB/IB in sheets	PB/IB (single or in small clusters in GC)	IB/CB	IB/PB/anaplastic	PB/IB (diffuse pattern of growth)
**CD20**	- (can be + in extracavitary PEL)	+/−	+/−	–	+ (− in cases with plasmacytic morphology)	Often +	- or weakly + in a minority of cells
**PAX5**	- (can be + in extracavitary PEL)	–	–	–	+	Often +	- or weakly + in a minority of cells
**CD79α**	- (can be + in extracavitary PEL)	−/+	–	–	+ (− in cases with plasmacytic morphology)	Often +	+ in about 40% of cases
**MUM1/IRF4**	+	+	+	+ (often)	+ in cases with plasmacytic morphology	Often +	+
**CD10**	–	–	–	–	–	–	- (rarely +)
**BCL6**	–	–	–	–	–	–	–
**BCL2**	–	–	–	–	–	+	–
**CD38**	+	−/+	−/+	+/−	–	–	+
**CD138**	+	–	–	–	+ in cases with plasmacytic morphology	−/+	+
**CD30**	+	–	- (rarely +)	+/−	Often +	−/+	+
**CD15**	–	–	–	–	–	–	–
**EMA**	Often +	–	–	–	–	–	+
**T cell markers**	Occasionally + (especially in extracavitary PEL)	–	–	Occasionally +	Occasionally +	–	Occasionally +
**Light chain restriction**	Usually absent	+ cIgM lambda	+ cIgM lambda	+ kappa or lambda	Often +	Often +	+ (often IgG kappa or lambda)
**HHV8**	+	+	+	+	–	–	–
**EBV (by LMP1)**	LMP1-	LMP1-	LMP1-	LMP1- (EBNA2-, BZLF-1-, type I EBV latency)	LMP1+ (EBNA1+, EBNA2+, type III EBV latency)	–	LMP1 -
**EBV (by EBER)**	+ (− in HIV-negative elderly pts. and in transplanted pts)	–	–	+	+	–	+ in 60–75% of cases
**Clonality**	Monoclonal (IG genes hypermutated). Rare MYC, BCL2, BCL6	polyclonal	Monoclonal (IgG genes unmutated)	Polyclonal or oligoclonal (rarely monoclonal)	Monoclonal (IG genes hypermutated)	Monoclonal (IG genes hypermutated) Frequent MYC, BCL2, BCL6 rearrangements	Monoclonal

*Legends*: *BM* bone marrow, *CB* centroblasts, *DLBCL-CI* diffuse large B-cell lymphoma associated with chronic inflammation, *EBV* Epstein Barr-virus, *EBER* in situ hybridization for EBV-encoded RNA, *GLPD* germinotropic lymphoproliferative disorder, *HD-like* Hodgkin-like, *HHV8+ MCD* HHV8-positive multicentric Castleman disease, *HHV8+ DLBCL* HHV8-positive diffuse large B-cell lymphoma, *HHV8- negative EBL* HHV8-negative effusion based lymphoma, *IB* immunoblasts, *LMP1* Latent membrane protein, *KS* kaposi sarcoma, *PB* plasmablasts, *PBL* plasmablastic lymphoma, *PEL* primary effusion lymphoma, *pts.* patients

HHV8-positive MCD commonly affects HIV-positive patients and rarely HIV-negative individuals from HHV8-endemic regions [1]. In the setting of SOT, mainly kidney, liver and heart transplantation, 11 cases of MCD have been reported so far [15]. HHV8-positive MCD shows systemic symptoms, multiple lymphadenopathy, cytopenia, splenomegaly, hypoalbuminemia, hypergammaglobulinemia, elevated serum levels of inflammatory markers such as C-reactive protein and interleukin 6 (IL-6) [1]. The disease usually shows an unfavorable outcome. The key histological features are regressed and hyalinized germinal centers (GCs) with prominent penetrating venules and interfollicular polytypic plasma cells [1]. Plasmablasts, isolated or in small aggregates, are seen within the mantle zone and interfollicular region. Plasmablasts, which are always IgM lambda positive, are commonly negative for B-cell markers (CD20, PAX5, CD79α) and CD138, and positive for CD38 and MUM1IRF4 [1]. MCD is usually EBV-negative and strongly associated with HHV8 infection, especially in HIV-positive cases. Despite monoclonal immunoglobulin, MCD often lacks monoclonal IGH gene rearrangement. In MCD, plasmablasts are scattered or in small clusters and, in a small number of cases, they may give rise to HHV8-positive DLBCL, NOS.

This aggressive lymphoma usually arises in HIV-positive individuals in the setting of HHV8-positive MCD and rarely de novo [1]. Recently two cases of HHV8-positive, DLBCL, NOS have been reported in renal transplant recipients [16]. Unlike PEL commonly involving body cavities without forming a mass, HHV8-positive DLBCL, NOS usually affects lymph nodes, spleen and liver; extranodal sites and bone marrow are often involved [1]. The architecture is totally effaced by sheets of plasmablasts. By definition, the neoplastic cells are HHV8-positive and EBV-negative [1]. The cells show the same MCD phenotype such as IgM lambda, MUM1/IRF4 and CD38 positivity and absence or weak expression of B-cell antigens, CD30 and CD138 [1]. Differentiating PEL, in its extracavitary variant, from HHV8-positive DLBCL, NOS can be difficult. PEL is often positive for CD138, EMA and CD30, in absence of cytoplasmic immunoglobulin. Unlike HHV8-positive DLBCL, NOS, PEL often shows HHV8 and EBV co-infection, especially in HIV- positive individuals. However, EBV infection is rarely identified in PEL affecting elderly as well as transplanted individuals. As mentioned above, the majority of PT-PEL, in which EBER was evaluated, were negative. This makes very tricky the differential diagnosis between PEL, in its extracavitary variant, and HHV8-positive DLBCL, NOS.

GLPD is a recent entity strictly linked to HHV8 infection, usually with an indolent behavior [1, 14]. It affects mainly immunocompetent individuals, with only rare cases described in HIV-positive patients [14]. GLPD has not been described in transplanted individuals. Patients are generally asymptomatic with localized lymphadenopathy. Aggregates of plasmablasts generally involving GCs of lymphoid follicles and co-infected by HHV8 and EBV are the key features [1, 14]. Plasmablasts are usually negative for B-cell markers (CD20, CD79α, PAX5) and CD138, with variable positivity for MUM1/IRF4, CD38 and CD30. An aberrant expression for T cell markers (CD3) can be present [17]. Plasmablasts often show monotypic kappa or lambda light chains, unlike MCD which is always IgM lambda positive. Despite monotypic immunoglobulin expression by plasmablasts, GLPD shows mainly a polyclonal pattern of IG gene rearrangement and rarely an oligoclonal or monoclonal pattern.

PEL, especially in its solid-form, can be misinterpreted as anaplastic large cell lymphoma (ALCL), due to the possible aberrant expression of T-cell markers together with CD30 positivity, however ALCL is always HHV8 negative. It is worth mentioning that in the case of PT-PEL positive for TCR gene rearrangement, a T-cell lymphoma was excluded due to HHV8 positivity [10].

DLBCL-CI is a mass-forming, EBV-driven aggressive neoplasm arising in the context of persistent chronic inflammation generally involving body cavities or narrow spaces [1]. The prototype of this category is pyothorax-associated lymphoma (PAL) which is a DLBCL occurring in patients who have undergone artificial pneumothorax as a therapy for tuberculosis and subsequently develop chronic pyothorax [1].

Unlike PEL classically not mass forming, in PAL patients present with a mass involving the pleura. The cells have a centroblastic or immunoblastic morphology and generally express B cell markers (CD20, CD79α); T-cell markers may be aberrantly expressed. CD30 can be positive [1]. A subset of cases with a plasmacytic morphology, lacking B cell markers and positive for plasma cell markers (MUM1/IRF4 and CD138) may be more difficult to differentiate from PEL. However, unlike PEL, DLBCL-CI is always HHV8 negative and EBV positive, with type III EBV latency pattern (positivity for LMP1 and EBNA2).

Recently, another EBV-positive and HHV8-negative entity has been included among DLBCL-CI, but renamed fibrin-associated diffuse large B-cell lymphoma (FA-DLBCL), because it develops within fibrinous material usually in the context of pseudocysts, cardiac myxoma, valve prosthesis, fibrin thrombus, synthetic tube graft, hydrocele, metallic implants and chronic subdural hematoma [1, 18, 19]. Differently from PAL which is mass-forming and follows an aggressive course, FA-DLBCL does not form masses and mostly has an indolent behavior often with the only surgery.

HHV8-negative EBL is a group of large B-cell lymphoma presenting as effusion without detectable tumor masses, a feature in common with typical PEL. Compared with PEL, in HHV8-negative EBL, patients are often older, HIV-negative and non-immunocompromised [20]. Some cases may be associated with a fluid overload status and hepatitis C infection. Morphologically the neoplastic cells are large with immunoblastic, plasmablastic or anaplastic fetaures, an overlapping morphology with PEL [20]. Unlike PEL showing a plasmablastic phenotype and lacking B-cell markers, the tumor cells of HHV8-negative EBL frequently express B-cell markers and less frequently CD138 and CD30. HHV8 is absent by definition. EBER is more frequently positive in classic PEL than in HHV8-negative EBL [20].

## Discussion

HHV8 is a gamma herpesvirus related to EBV. HHV8 was initially identified in KS and named KS-herpesvirus (KSHV) [21]. Later, HHV8 was identified to be the causative agent of MCD [22] and PEL [23]. Most HHV8-associated diseases occur in immunocompromised individuals, with the exception of GLPD [1, 14]. HHV8 is not ubiquitous in the worldwide population. There are geographic areas such as sub-Saharan Africa, Latin America, Carabbean, Mediterranean and Middle Eastern countries where HHV8 infection is endemic and there is a known high incidence of classic/endemic KS [24]. Transmission of virus occurs through saliva, but it may be transmitted sexually, vertically through breast milk, by intravenous drug use, blood transfusion and through transplant [25]. As EBV, HHV8 may infect lymphoid cells and other cell types like endothelial cells and persist lifelong in a latent form [25]. When the immune control decreases, HHV8 may reactivate the lytic replicative cycle producing viremia. In post-transplant patients, iatrogenic immunosuppression may be the cause of HHV8 reactivation, leading to uncontrolled expansion of latently infected endothelial cells or mature post-germinal center B cells [25]. HHV8-related diseases occurring in the setting of transplantation may be the result of reactivation of a pre-existing HHV8 infection in the recipient host or of HHV8 transmission from HHV8-seropositive donors. The risk to develop post-transplant HHV8-related diseases depends on the ethnic origin of both the donor and the recipient. Given the lack of standardization and variable sensitivity and specificity, HHV8 serologic testing is not routinely included in pre-transplant screening [25]. Despite being the seroconversion rate among seronegative SOT transplant recipients from seropositive donors quite high (30% by indirect immunofluorescence), the development of HHV8 viremia and HHV8-driven diseases is much less frequent. For this reason, screening SOT recipients and donors for HHV8 to assess the risk of post-transplant HHV8-related diseases is not used in routine practice [25].

Post-transplant lymphoproliferative disorders (PTLDs) represent a rare, but potentially fatal complication related to iatrogenic immunosuppression. According to the current WHO classification, PTLDs include a wide spectrum of diseases ranging from non-destructive lymphoid proliferation to overt lymphomas [1]. The association between EBV and PTLDs is well recognized [1], whereas the role of HHV8 in post-transplant malignancy is less familiar, despite a high incidence of KS among transplant recipients especially in endemic regions [25]. Post-transplant HHV8-driven pathologies are rare and include neoplastic proliferations like KS, MCD, PEL and HHV8-positive DLBCL, NOS. PT-KS is the most commonly reported HHV8-related disease after SOT, developing in up to 1% of organ recipients, mainly kidney recipients, whereas it is rarely seen after haematopoietic stem cell transplantation (HSCT), probably as a consequence of damaged HHV8 cell reservoirs [25]. PT-MCD is a rare complication of transplantation, occurring in less than 1% of SOT. Concomitant MCD and KS may develop in organ recipients [15]. Recently, Gwiti et al. [16] reported the first two cases of HHV8-positive DLBCL, NOS in transplanted patients. Both cases were diagnosed at autopsy with synchronous KS and showed an uncommon intravascular pattern of growth.

In addition to frank neoplastic diseases, in transplanted patients HHV8 may be the etiologic agent of serious non-neoplastic disorders typically associated with high HHV8 viremia. These non-neoplastic disorders include the usually polyclonal, but often fatal Castleman-like or atypical HHV8-positive plasmacytic lymphoproliferations involving lymph nodes and visceral organs; acute bone marrow failure often with signs of hemophagocytic syndrome; and acute hepatitis syndromes [26].

PEL is a rare aggressive B-cell lymphoma affecting a unique population of individuals who are often immunocompromised or elderly. In 1995 Cesarman et al, linked PEL etiologically to HHV8 infection [23]. PEL may exhibit coinfection with EBV, especially in HIV-positive patients. It commonly presents as malignant effusions of body cavities, although an extracavitary variant of PEL presenting with tumor masses is described [1].

In 1995, Jones et al reported the first case of fatal PT-PEL occurring 94 months after heart transplantation [2]. Like EBV, post-transplant HHV8 infection can be acquired by an HHV8-negative recipient of an organ from an infected donor or may represent autoreactivation of a latent pre-existing HHV8 infection in the recipient. It has been suggested that in recipients from countries with high HHV8 serological prevalence, the risk of PT-KS is mainly due to HHV8 reactivation rather than to organ-transmitted primary HHV8 infection [25, 26]. The same phenomenon may be hypothesized for PT-PEL.

PT-PEL is a rare and life-threatening complication mainly occurring in SOT recipients. Our literature search identified 13 cases of PT-PEL so far [2–13]. PT-PEL was reported in 6 renal, 3 heart, 2 liver and 1 intestine recipients. Only one case of PT-PEL occurred in an allogenic bone marrow recipient [7]. Like PT-PEL, PT-KS is more frequently observed in kidney recipients, with smaller incidence in recipients of other organs, mainly heart and liver, whereas it is a rather uncommon event in bone marrow (BM) recipients [27]. It has been hypothesized that myeloablative conditioning chemotherapy and total body irradiation may destroy HHV8-positive cells reservoir in BM recipients, while immunosuppressive regimens used in SOT do not destroy the cells harboring latent HHV8, which may reactivate [27]. Another explanation for the rarity of KS and PEL occurrence after BM transplant is the protective effect given by the abnormal inflammatory conditions associated with immune reconstitution [27]. KS occurred in association with PEL in 4 SOT recipients, preceding the occurrence of PEL in three cases [2, 4, 12]. PT-PEL generally showed the classical PEL presentation with body cavity effusions in absence of tumor masses. Cain et al [11], reported a case of PT-PEL in an HIV-positive individual presenting with effusions associated with multiple organ involvement. Two cases of extracavitary PT-PEL presenting with solid masses, in absence of body cavities involvement, were described by Testa et al [6] and Zanelli et al [13].

Unlike EBV-associated PTLDs in which the risk is higher in the early post-transplant period and declines over time, long-term immunosuppression is important in PT-PEL commonly developing several years after transplantation. The diagnosis of PT-PEL relies on the above described clinical and histopathological features along with HHV8 immunohistochemical demonstration. EBV coinfection is usually absent in PT-PEL, unlike in HIV-positive PEL patients.

PT-PEL has a poor prognosis. Most of the reported cases with available follow-up died in less than 1 year. In HIV-positive patients, PEL is treated with systemic chemotherapy (frequently CHOP-based regimen) along with antiretroviral therapy. In PT-PEL, in addition to chemotherapy, reduction of immunosuppressive treatment, is generally attempted. An impressive regression of PT-KS has been reported switching immunosuppression from calcineurin inhibitors to mammalian target of rapamycin (mTOR) inhibitors, such as sirolimus with broad antineoplastic activity in vitro [25]. So far, PT-PEL do not appear to benefit from sirolimus, as suggested by 2 cases of PT-PEL occurring under sirolimus [4]. However, Boulanger et al suggested that the use of sirolimus at the dose recommended to avoid rejection may not be sufficient to prevent the occurrence of PEL [4]. Kugasia et al reported a case of PT-PEL, in which, in adjunct to chemotherapy, tacrolimus was changed to sirolimus leading to undetectable HHV8 viremia [12]. Based on the limited number of cases, a switch to sirolimus should not be discouraged in PT-PEL, though no evidence of efficacy currently exists. CHOP-based chemotherapy still remains a therapeutic option for the treatment of PT-PEL patients, who are often refractory to modifications of immunosuppressive therapy and intravenous antivirals. Recent data support the possible role of anti-CD30 monoclonal antibody brentuximab in the treatment of PEL. The case by Kugasia et al constitutes the first reported use of Brentuximab for post-SOT PEL [12]. The patient was treated with brentuximab in adjunct to cyclophosphamide, vincristine and prednisone and immunosuppression was changed from tacrolimus to sirolimus. The patient responded well to therapy with no recurrence of effusions and viremia; the cause of death was unrelated to PEL. So far, in PT-PEL chemotherapy is recommended in association with reduction of immunosuppression (if possible) and antivirals (particularly if high viral load) [26]. In case of chemotherapy failure, the use of antiviral drugs such as cidofovir either systemic or intracavitary as salvage therapy is considered [26].

## Conclusions

In immunosuppressed organ recipients, HHV8 is responsible for several virus-driven diseases, of which PEL represents a rare, but life-threatening complication. It is possible that a proportion of HHV8-related diseases, including PT-PEL, could be missed as the diagnosis is often difficult and the cases reported too few. Clinicians should suspect PEL particularly in SOT recipients presenting with unexplained body cavity effusions. The relative infrequency of HHV8-driven diseases together with the difficulty in achieving a diagnosis and the mostly unfavorable outcome support the need for a deeper understanding.

## Data Availability

Not applicable as no datasets were generated or analyzed during the current study.

## Abbreviations

ALCL: Anaplastic Large Cell Lymphoma
ASCT: Allogenic Stem Cells Transplantation
BM: Bone Marrow
CHOP: Cyclophosphamide, doxorubicin, prednisone, vincristine
DLBCL-CI: Diffuse Large B-cell Lymphoma associated with Chronic Inflammation
EBER: Epstein-Barr virus-Encoded small RNA
EBL: Effusion Based Lymphoma
EBNA2: Epstein-Barr Nuclear Antigen 2
EBV: Epstein-Barr Virus
EMA: Epithelial Membrane Antigen
FA-DLBCL: Fibrin-Associated Diffuse Large B-Cell Lymphoma
GCs: Germinal Centers
GLPD: Germinotropic Lymphoproliferative Disorder
GVHD: Graft Versus Host Disease
HAART: Highly Active Anti-Retroviral Therapy
HHV8: Human Herpes-Virus 8
HHV8-positive DLBC, NOS: Human Herpes Virus 8-positive Diffuse Large B-Cell Lymphoma, Not Otherwise Specified
HIV: Human Immunodeficiency Virus
HLA-DR: Human Leucocyte Antigen-DR isotype
HSCT: Haematopoietic Stem Cells Transplantation
IG: Immunoglobulin
IGH: Immunoglobulin Heavy chain
IGL: Immunoglobulin Light chain
IL-6: InterLeukin 6
KS: Kaposi Sarcoma
KSHV: Kaposi Sarcoma Herpes Virus
KSHV/HHV8: Kaposi Sarcoma Herpes Virus/Human Herpes Virus 8
LANA-1: Latency-Associated Nuclear Antigen
LCA: Leucocyte Common Antigen
LMP1: Latent Membrane Protein 1
MCD: Multicentric Castleman disease
MeSH: Medical Subject Headings
mTOR: Mammalian Target Of Rapamycin
MUM1/IRF4: Multiple Myeloma1/Interferon Regulatory Factor 4 protein
PAL: Pyothorax Associated Lymphoma
PCR: Polymerase Chain Reaction
PEL: Primary Effusion Lymphoma
PRISMA: Preferred Reported Items for Systematic Reviews and Meta-Analyses
PT-KS: Post-transplant Kaposi Sarcoma
PTLDs: Post-Transplant Lymphoproliferative Disorders
PT-MCD: Post-transplant Multicentric Castleman Disease
PT-PEL: Post-Transplant Primary Effusion Lymphoma
SOT: Solid Organ Transplant
TCR: T-Cell Receptor
WHO: World Health Organization
